# Ultra-High Adsorption Capacity of Core–Shell-Derived Magnetic Zeolite Imidazolate Framework-67 as Adsorbent for Selective Extraction of Theophylline

**DOI:** 10.3390/molecules28145573

**Published:** 2023-07-21

**Authors:** Ling-Xiao Chen, Shi-Jun Yin, Tong-Qing Chai, Jia-Li Wang, Guo-Ying Chen, Xi Zhou, Feng-Qing Yang

**Affiliations:** School of Chemistry and Chemical Engineering, Chongqing University, Chongqing 401331, China; 20175534@cqu.edu.cn (L.-X.C.); 201718021124@cqu.edu.cn (S.-J.Y.); 20175531@cqu.edu.cn (T.-Q.C.); 202118021010@cqu.edu.cn (J.-L.W.); 20221801017@stu.cqu.edu.cn (G.-Y.C.); 202018021119@cqu.edu.cn (X.Z.)

**Keywords:** magnetic zeolite imidazolate framework, core–shell-derived structure, theophylline, magnetic solid-phase extraction, tea

## Abstract

A core–shell-derived structural magnetic zeolite imidazolate framework-67 (Fe_3_O_4_-COOH@ZIF-67) nanocomposite was fabricated through a single-step coating of zeolite imidazolate framework-67 on glutaric anhydride-functionalized Fe_3_O_4_ nanosphere for the magnetic solid-phase extraction (MSPE) of theophylline (TP). The Fe_3_O_4_-COOH@ZIF-67 nanocomposite was characterized through scanning electron microscopy, transmission electron microscopy, energy dispersive X-ray spectrometry, Fourier transform infrared spectroscopy, Zeta potential analysis, X-ray diffraction, Brunauer–Emmett–Teller, and vibrating sample magnetometer. The material has a high specific surface area and good magnetism, which maintains the regular dodecahedron structure of ZIF-67 without being destroyed by the addition of Fe_3_O_4_-COOH nanospheres. The Fe_3_O_4_-COOH@ZIF-67 can rapidly adsorb TP mainly through the strong coordination interaction between undercoordinated Co^2+^ on ZIF-67 and –NH from imidazole of TP. The adsorption and desorption conditions, such as the amount of adsorbent, adsorption time, pH value, and elution solvent, were optimized. The kinetics of TP adsorption on Fe_3_O_4_-COOH@ZIF-67 was found to follow pseudo-second-order kinetics. The Langmuir model fits the adsorption data well and the maximum adsorption capacity is 1764 mg/g. Finally, the developed MSPE-HPLC method was applied in the enrichment and analysis of TP in four tea samples and rabbit plasma. TP was not detected in oolong tea and rabbit plasma, and its contents in jasmine tea, black tea, and green tea are 5.80, 4.31, and 1.53 μg/g, respectively. The recoveries of spiked samples are between 74.41% and 86.07% with RSD in the range of 0.81–3.83%. The adsorption performance of Fe_3_O_4_-COOH@ZIF-67 nanocomposite was nearly unchanged after being stored at room temperature for at least 80 days and two consecutive adsorption–desorption cycles. The results demonstrate that Fe_3_O_4_-COOH@ZIF-67 nanocomposite is a promising magnetic adsorbent for the preconcentration of TP in complex samples.

## 1. Introduction

Theophylline (1,3-dimethyl-7*H*-purine-2,6-dione) (TP) is a dimethylxanthine derived from the xanthine purine base of alkaloid, which occurs naturally in most kinds of teas (jasmine tea, black tea, oolong tea, etc.) and cocoa beans in trace amounts [1,2,3]. TP is an important bioactive component of tea, which has various bioactivities at low doses [4]. TP can relax the airway smooth muscle mainly through the inhibition of phosphodiesterase (PDE)3 activity [1]. It is widely used to treat airway obstruction caused by clinical conditions such as asthma, infant apnea, chronic bronchitis, emphysema, and chronic obstructive pulmonary disease [5,6,7]. In recent years, TP has also been proposed and used as a supplement to treat patients with COVID-19 in 2019 [8]. Therefore, effective separation and reliable quantification of TP are becoming increasingly attractive.

High-performance liquid chromatography coupled with mass spectrometry (HPLC-MS) has been employed to determine TP from various tea and biological samples [3]. However, the direct determination of TP in tea and biological samples is always faced with complicated matrix interferences and trace analytes. Hence, the sample pretreatment procedure to separate and enrich TP from complex samples is indispensable before chromatographic analysis. In recent years, some methods, including liquid–liquid extraction (LLE) [9], electromembrane extraction (EME) [10], pipette-tip solid-phase extraction (PT-SPE) [11], ultrasound-assisted surfactant-enhanced emulsification microextraction (UA-SEME) [12], dispersive liquid–liquid microextraction (DLLME) [13], and solid-phase extraction (SPE) [14] have been reported. However, there are some disadvantages of these methods, derived from the low extraction capacity, complicated procedures, and consumption of toxic organic solvents. Magnetic solid-phase extraction (MSPE), which is an easy and quick pre-enrichment procedure for concentrating target analytes from complex samples by applying an external magnetic field [15], has attracted much interest owing to its low cost, fast separation, high efficiency, and selectivity [16]. The choice of a suitable adsorbent is vital in MSPE and depends primarily on the nature of the sample being tested [17]. To date, MSPE has been reported to perform selective extraction of TP from tea and plasma samples, using molecular-imprinted polymers (MIP) [18], copper-doped magnetic microspheres [19], and metal–organic framework (MOF) [20] as magnetic adsorbents. However, these absorbents have a limited adsorption capacity.

MOF, also recognized as a porous crystalline polymer (PCP), is regarded as a typical crystalline porous material, which can be easily self-assembled by metal ions and/or clusters as a secondary building unit connected with different organic ligands through various coordination bonds [21]. MOFs and their derivatives have been well studied for different applications, such as gas storage and separation [22], gas removal [23], pollutant removal [24], heterogeneous catalysis [25], drug delivery [26], sensing [27], and energy storage and conversion [28], due to their low diversity, tunable pore size, large surface area, and adjustable structure [29]. As a typical type of MOF, zeolitic imidazolate frameworks (ZIFs) are composed of Zn^2+^ or Co^2+^ and imidazolate linkers [30]. ZIF-67, which is composed of Co^2+^ and 2-methylimidazole, has a sodalite-like topological structure, and each unit cell contains two sodalite cages. The diameter of the sodalite cage is 11.4 Å, and each sodalite cage is connected by a six-membered ring cage composed of six Co. The six-membered ring cage has a diameter of 3.4 Å [31]. ZIF-67 has been successfully used as an SPE adsorbent to concentrate different compounds, such as antibiotics [32], insecticides [33], and dyes [34], due to its excellent thermal and chemical stability in water, as well as alkaline and acidic solutions. Therefore, magnetic ZIF-67 can be used as an adsorbent in MSPE, and there are different approaches have been reported for the magnetization of MOF materials: (1) Direct magnetization [35], which is obtained through direct mixing of the MOF and magnetic NPs in a specific solution with stirring or sonication. This method is a simple operation and has good magnetic properties, but a relatively small specific surface area leads to a low extraction efficiency. (2) In situ growth of magnetic NPs [36], which is based on the prepared MOF, followed by dispersion in the precursor solution of the Fe_3_O_4_ NPs, but the original morphology of MOF will be destroyed. (3) Single-step MOF coating [37], the prepared magnetic NPs with proper modifications are added to a solution containing the precursors of MOF, which can maintain the maximum degree of morphology of MOF, having a large specific surface to obtain a high adsorption capacity. (4) Layer-by-Layer (LbL) MOF growth [38], which repeats the process of the single-step MOF coating method, is time-consuming and the magnetism of obtained MOF is weak. (5) MOF carbonization under an inert atmosphere [39]. In this method, the MOF is used as a precursor material calcined at high temperature under vacuum to produce magnetic porous carbon, which requires a high-temperature treatment, and the original MOF is destroyed completely. Therefore, to maintain the original morphology and high specific surface area of ZIF-67, the single-step MOF coating method may be one of the best choices to prepare the related magnetic materials.

**Figure 1:**
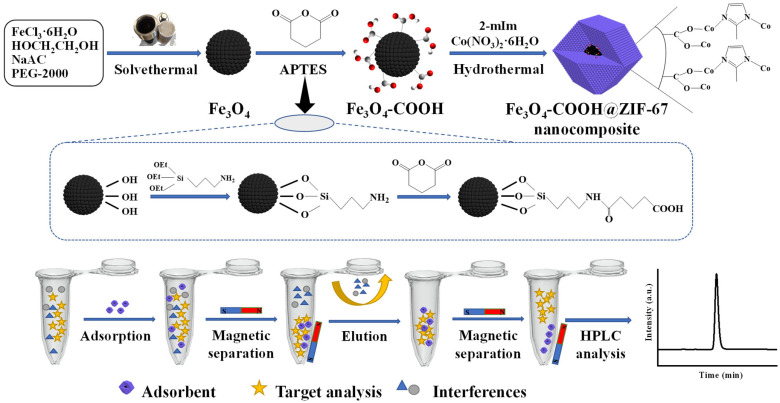
Schematic diagram of the preparation procedure of Fe_3_O_4_-COOH@ZIF-67 and MSPE process.

In this study, a novel core–shell-derived structural magnetic Fe_3_O_4_-COOH@ZIF-67 nanocomposite was fabricated through a single-step coating of ZIF-67 on glutaric anhydride-functionalized Fe_3_O_4_ nanosphere, which was prepared firstly using a solvothermal method [32]. The surface modification of Fe_3_O_4_ nanoparticle by glutaric anhydride can provide a carboxyl group to coordinate with the Co^2+^ of ZIF-67, which was used as a core–shell linker for Fe_3_O_4_ and ZIF-67 [40]. The core–shell-derived material was systematically characterized, and the parameters of adsorption and desorption for TP were optimized. Furthermore, the adsorption behavior and mechanism were illustrated by adsorption isotherms and kinetic studies. Finally, the prepared Fe_3_O_4_-COOH@ZIF-67 nanocomposite was employed as the magnetic adsorbent for the enrichment and analysis of TP in four tea samples (jasmine tea, black tea, green tea, and oolong tea) and rabbit plasma. The flow diagram of the MSPE procedure is shown in Figure 1.

## 2. Results and Discussion

### 2.1. Characterizations of the Prepared Materials

**Figure 2:**
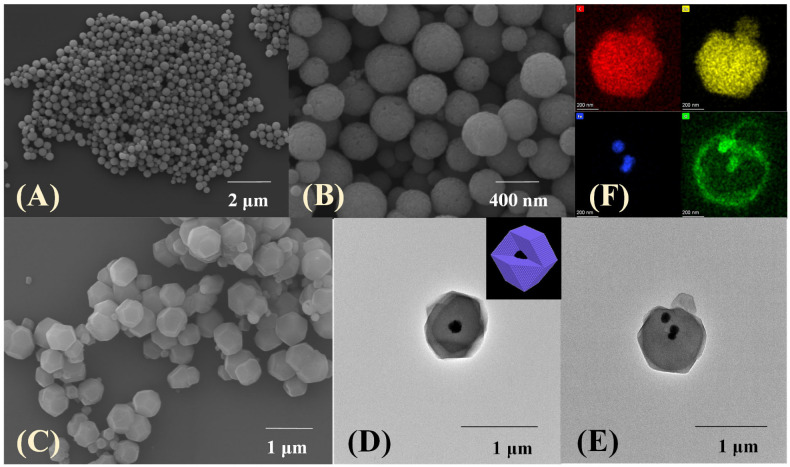
The SEM images of (**A**) Fe_3_O_4_, (**B**) Fe_3_O_4_-COOH, and (**C**) Fe_3_O_4_-COOH@ZIF-67; (**D**,**E**) TEM images of Fe_3_O_4_-COOH@ZIF-67; (**F**) EDX elemental mapping analysis images of Fe_3_O_4_-COOH@ZIF-67.

**Figure 3:**
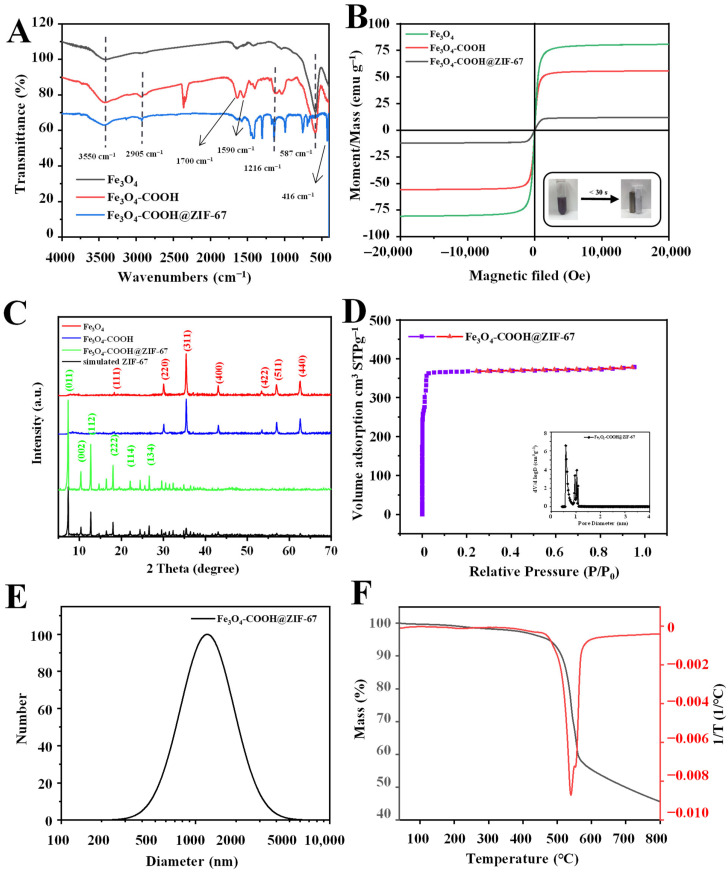
(**A**) FT-IR spectra and (**B**) magnetization curves of Fe_3_O_4_, Fe_3_O_4_-COOH, and Fe_3_O_4_-COOH@ZIF-67; (**C**) XRD patterns of Fe_3_O_4_, Fe_3_O_4_-COOH, Fe_3_O_4_-COOH@ZIF-67, and ZIF-67; (**D**) nitrogen adsorption–desorption isotherms and pore size distribution; (**E**) DLS particle diameter distribution; and (**F**) TGA curve of Fe_3_O_4_-COOH@ZIF-67.

The surface morphology of the prepared materials was investigated through scanning electron microscopy (SEM) and transmission electron microscopy (TEM). The average diameter of pure Fe_3_O_4_, which is uniform in shape and size (Figure 2A), is mainly distributed in the range of 250–350 nm. A rougher spherical surface and slightly increasing diameter can be observed after being functionalized by glutaric anhydride (Figure 2B). Furthermore, the Fe_3_O_4_-COOH nanosphere is embedded in the core–shell-derived composite structure and the ZIF-67 crystal (~300 nm thickness) is served as the shell, and the mean particle size of Fe_3_O_4_-COOH@ZIF-67 is about 900 nm (Figure 2C). Meanwhile, the hydrodynamic particle size of Fe_3_O_4_-COOH@ZIF-67 (Figure 3E), as measured by dynamic light scattering (DLS), is about 1100 nm. The prepared Fe_3_O_4_-COOH@ZIF-67 is monodispersed and without aggregation in aqueous solution. Figure 2D,E reveals that Fe_3_O_4_-COOH@ZIF-67 nanocomposite maintains the regular dodecahedron structure of ZIF-67 without being destroyed by the addition of Fe_3_O_4_-COOH nanospheres. In addition, energy-dispersive X-ray spectroscopy (EDX) mapping analysis images in (Figure 2F) prove the existence of C, O, Co, and Fe in the Fe_3_O_4_-COOH@ZIF-67 nanocomposite and all element signals are localized homogeneously. These results reveal the successful preparation of Fe_3_O_4_-COOH@ZIF-67 nanocomposite.

The functional groups on the surface of prepared materials were characterized using Fourier transform infrared (FT-IR) spectroscopy, and the results are shown in Figure 3A. For Fe_3_O_4_, the strong absorption peak at 587 cm^−1^ is assigned to the Fe–O bond stretching vibration. For Fe_3_O_4_-COOH, the absorption peaks observed at 3550 cm^−1^, 2905 cm^−1^, and 1700 cm^−1^ correspond to the stretching vibration of O-H, C-H, and C=O bonds, respectively. Moreover, the asymmetric stretching vibration of COO^−^ can be observed at 1590 cm^−1^. These results can prove the successful functionalization of glutaric anhydride on the surface of the Fe_3_O_4_ nanosphere. For Fe_3_O_4_-COOH@ZIF-67, new absorption peaks between 995 and 1380 cm^−1^ and at 1417 cm^−1^ are attributed to the plane bending and stretching of the imidazole ring [37], respectively. In addition, a peak corresponding to the Co-N stretching vibration can be observed at 416 cm^−1^ [41]. However, the characteristic absorption peak at 587 cm^−1^ related to the Fe-O stretching vibration of Fe_3_O_4_ disappeared, which confirms that the Fe_3_O_4_ nanospheres are embedded into the Fe_3_O_4_-COOH@ZIF-67 rather than on its surface.

The magnetic characteristics of Fe_3_O_4_, Fe_3_O_4_-COOH, and Fe_3_O_4_-COOH@ZIF-67 were investigated through a vibrating sample magnetometer (VSM) at room temperature. As shown in Figure 3B, their hysteresis loop is S-type, and no obvious remanence or coercivity is observed, indicating that the as-prepared materials exhibit typical super-paramagnetic behaviors. The saturation magnetization values of Fe_3_O_4_ and Fe_3_O_4_-COOH are 80.68 emu g^−1^ and 55.76 emu g^−1^, respectively. The saturation magnetization value of Fe_3_O_4_-COOH@ZIF-67 is 12.14 emu g^−1^, due to the presence of glutaric anhydride layer and ZIF-67 shell. Although the magnetic saturation value of the adsorbent is relatively low, it is enough to separate the adsorbent from the sample solution within 30 s (inset of Figure 3B).

The X-ray diffraction (XRD) analysis was performed to further investigate the crystal structure of Fe_3_O_4_-COOH@ZIF-67 nanocomposites, and the results are shown in Figure 3C. The diffraction peaks of the three prepared materials are sharp and intense, indicating that they are highly crystalline. The diffraction peaks at 30.2° (220), 35.5° (311), 43.2° (400), 53.6° (422), 57.1° (511), and 62.7° (440) are consistent with the Bragg diffraction pattern of pure Fe_3_O_4_. In addition, the peak intensity of Fe_3_O_4_ is weaker after glutaric anhydride functionalization, but the crystal structure of Fe_3_O_4_ remains unchanged. After the introduction of ZIF-67 (Fe_3_O_4_-COOH@ZIF-67), new diffraction peaks can be observed at 7.2° (011), 10.2° (002), 12.5° (112), 14.5° (022), 16.4° (013), 17.8° (222), 24.2° (114), 26.4° (134), and 29.5° (044) [42], respectively, which are similar to that of the simulated XRD pattern of ZIF-67. However, the diffraction peaks of Fe_3_O_4_ nearly vanished. These results prove the successful preparation of core–shell-derived structure Fe_3_O_4_-COOH@ZIF-67 and that the ZIF-67 can retain its original morphology.

The pore structure parameters (Table S1) of Fe_3_O_4_-COOH@ZIF-67 were analyzed through Brunauer–Emmett–Teller (BET), and nitrogen adsorption–desorption isotherms (Figure 3D) were obtained at 77 k. The pore size distribution curve of Fe_3_O_4_-COOH@ZIF-67 shows the average pore diameter of 1.5983 nm calculated through the Barrett–Joyner–Halenda (BJH) method, and bimodal pore size (calculated through the Horvath–Kawazoe method) distribution centering at 5.88 and 9.88 Å (Figure 3D inset), indicating that TP can be adsorbed on both the pore channel and the surface of the adsorbent. Furthermore, Fe_3_O_4_-COOH@ZIF-67 displays a type I isotherm, suggesting its main microporous structure. In addition, Fe_3_O_4_-COOH@ZIF-67 shows a high BET-specific surface area (1465.3 m^2^/g) and pore volume (0.5855 cm^3^/g), which are the important reasons for its ultra-high adsorption capacity.

The obtained absorbent can be well dispersed in methanol to form a suspension and kept for several months without settlement. The thermal property of the obtained Fe_3_O_4_-COOH@ZIF-67 was investigated through thermogravimetric analysis (TGA). The TGA curve (Figure 3F) of Fe_3_O_4_-COOH@ZIF-67 shows that only 3.87% weight loss up to 340 °C, relating to the evaporation of guest molecules from cavities or unreacted species trapped within pores or the framework. Furthermore, Fe_3_O_4_-COOH@ZIF-67 has obvious weight loss (about 41.67%) at 340–560 °C, which is due to the collapse of the ZIF-67 framework. In addition, the weight loss (8.65%) at 560–800 °C is related to the decomposition of glutaric anhydride and the carbonization of the remains. These experimental results prove the existence of ZIF-67. At 800 °C, the residue (45.78%) of Fe_3_O_4_-COOH@ZIF-67 may be attributed to the thermal resistance of Fe_3_O_4_ particles.

### 2.2. Optimization of Experimental Conditions of MSPE

To obtain the optimum extraction performance, several adsorption–desorption conditions were investigated and optimized (50 μg/mL of TP and volume of 2 mL), including the amount of adsorbent, adsorption time, pH value, ion strength, and temperature (adsorption conditions), as well as type, pH, concentration and volume of elution solvent, and elution time (desorption conditions). Finally, ultrasound for 5 min before adsorption, 1.0 mg of adsorbent, pH 6, no PO_4_^3−^ addition, 30 °C, and adsorption of 20 min were selected for the adsorption step, and 3 mL of Na_3_PO_4_ (50 mM, pH 12) and desorption of 5 min were selected for desorption step.

#### 2.2.1. Amount of Adsorbent

**Figure 4:**
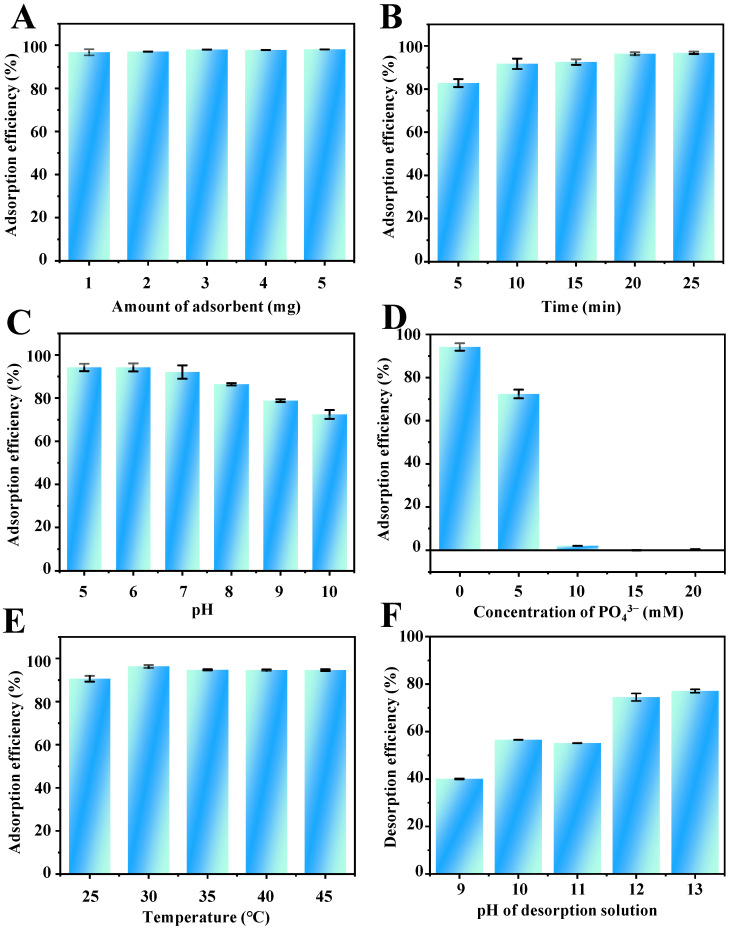
Effects of (**A**) amount of adsorbent, (**B**) adsorption time, (**C**) pH value, (**D**) ionic strength (PO_4_^3−^), (**E**) temperature on the adsorption efficiency of TP, and (**F**) the pH of desorption solvent on the desorption efficiency of TP.

In the MSPE procedure, the amount of adsorbent is one of the critical conditions that affect the extraction recovery. To obtain a satisfactory extraction performance, the amount of adsorbent was studied in the range of 1.0–5.0 mg. The results shown in Figure 4A suggest that the adsorption efficiency of TP barely changed with the increasing amount of Fe_3_O_4_-COOH@ZIF-67. Therefore, 1.0 mg of Fe_3_O_4_-COOH@ZIF-67 was selected in the following experiments.

#### 2.2.2. Adsorption Time

Rational adsorption time is essential to obtain adsorption equilibrium between analytes and adsorbents. Therefore, the effect of adsorption time on the adsorption efficiency of TP was investigated from 5 to 25 min. As shown in Figure 4B, when the adsorption time is extended to 20 min, the adsorption efficiency reaches a plateau value. The rapid adsorption process can be attributed to the high specific surface area and multiple strong interactions between the analytes and adsorbent. Therefore, 20 min was selected for achieving satisfactory adsorption efficiency and rapid analysis of TP.

#### 2.2.3. Adsorption pH

The pH of the sample solution can affect the electrostatic and coordination interaction between adsorbents and target analytes. Electrostatic interactions between TP and Fe_3_O_4_-COOH@ZIF-67, and coordination between Co^2+^ on the adsorbent surface and –NH group at position 9 of the TP imidazole ring, may strongly influence the extraction efficiency of TP. However, Fe_3_O_4_-COOH@ZIF-67 will gradually disintegrate when the pH value is lower than 3.0 from the experimental phenomena. Therefore, the effect of sample pH on the adsorption efficiency of TP was studied within the range of 5.0–10.0. As shown in Table S2, the structure of TP contains an imidazole ring (pK_a1_ = 1.6, pK_a2_ = 8.6). Thus, the charge of TP can be positive at pH < pK_a1_, zwitterionic in the range of pK_a1_ to pK_a2_, and negative at pH > pK_a2_. As shown in Figure 4C, the adsorption efficiency of TP is increased slowly with increasing pH from 5.0 to 6.0 and then decreased obviously from pH 6.0 to 10.0. Thus, the highest adsorption efficiency of TP was obtained at pH 6.0. Figure S1D presents the Zeta potential of adsorbent at varying pH values from 5 to 10. The zero point of charge (pH_ZPC_) for Fe_3_O_4_-COOH@ZIF-67 is at pH = 5.72. Therefore, the adsorbent surface is negatively charged when the pH of the solution is >5.72 and positively charged when the pH is <5.72. With the increase in the pH value of the solution to 5.72, the TP molecule is deprotonated and gradually negatively charged. In this regard, the electrostatic repulsion is weakening and gradually transforming to electrostatic attraction, leading to the enhancement in the adsorption efficiency of TP. When 5.72 < pH < 10, the surface of Fe_3_O_4_-COOH@ZIF-67 changes gradually from positively charged to negatively charged but the TP molecule is negatively charged, the electrostatic attraction is weakening, leading to the continuous declination in the adsorption efficiency of TP. Meanwhile, the declination may be the result of decreasing Co^2+^ positive charge on the sorbent surface, which weakens the coordination interaction between –NH on TP and Co^2+^ cations. Therefore, the sample solution pH value of 6.0 was employed for the follow-up experiments.

#### 2.2.4. Ionic Strength

The influence of ionic strength on the adsorption efficiency of TP was investigated with the NaH_2_PO_4_ concentration range from 0 to 20 mM. As shown in Figure 4D, the adsorption efficiency of TP decreases dramatically to zero with the increase in NaH_2_PO_4_ concentration. The viscosity of the aqueous phase will increase with the addition of salt ions, resulting in a difficult mass transfer of TP from aqueous solution to adsorbent [43]. On the other hand, according to the Hard Soft Acids Bases (HSAB) theory [44], PO_4_^3−^ can be classified as hard Lewis bases rather than an imidazole ring on the TP, which tends to compete with Co^2+^ on Fe_3_O_4_-COOH@ZIF-67 through coordination. Furthermore, the results also indicate that the interaction between the target compound and the adsorbent is controlled by the intermolecular interaction forces, such as hydrogen bond interactions, van der Walls forces, and π–stacking, which are easily affected by the counterbalance of ion concentration in the solution. Therefore, the samples without the addition of salts were used in the subsequent experiments.

#### 2.2.5. Adsorption Temperature

Adsorption temperature is one of the most critical factors affecting the adsorption efficiency. The increase in adsorption temperature can improve the mass transfer rate and fasten the extraction equilibrium process. On the other hand, from a thermodynamic point of view, since most adsorption processes are exothermic, the equilibrium adsorption capacity may decrease with increasing temperature. In this study, the influence of temperature on the adsorption efficiency of TP by Fe_3_O_4_-COOH@ZIF-67 was investigated at a temperature of 25–45 °C. As illustrated in Figure 4E, the adsorption efficiency of TP exhibits a remarkable increase from 25 to 30 °C and is almost constant with a further increase in temperature. Thus, 30 °C was selected as the optimum adsorption temperature.

#### 2.2.6. Desorption Conditions

The effects of six different elution solvents, including ethanolamine, diethanolamine, triethanolamine, Na_3_PO_4_, NH_3_·H_2_O, and Na_2_HPO_4_ on the desorption efficiency were compared. The result (Figure S1A) indicates that Na_3_PO_4_ aqueous solution (pH 11.7) has the best desorption efficiency. This may be due to the fact that PO_4_^3−^ can competitively coordinate with Co^2+^ on ZIF-67, causing the desorption of TP. Then, the effect of the Na_3_PO_4_ aqueous solution of different pH values (9–13) on the desorption efficiency was investigated. As shown in (Figure 4F), 12.0 is the best pH value. The coordination interaction and hydrogen bonding between TP and adsorbent can be destroyed by the high concentration of OH^−^ in a strong alkali solution. Furthermore, the volume and concentration of elution solvent were selected as 3.0 mL and 50 mM of Na_3_PO_4_, respectively (Figure S1B,C). In addition, 5 min is enough to elute the TP from the Fe_3_O_4_-COOH@ZIF-67.

### 2.3. Extraction Selectivity on TP of Fe_3_O_4_-COOH@ZIF-67

**Figure 5:**
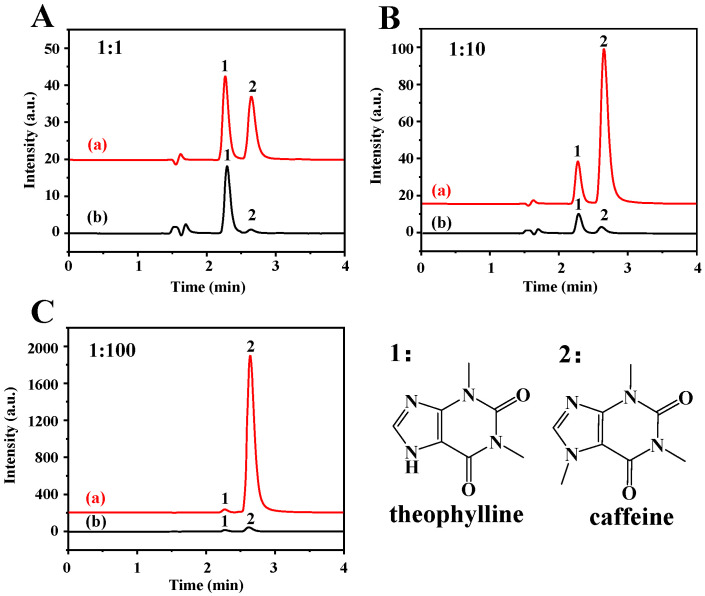
HPLC chromatograms of (a) mixture of TP and caffeine, and (b) elution solution after MSPE. The concentration ratios of TP and caffeine are (**A**) 1:1, (**B**) 1:10, and (**C**) 1:100, respectively. Peak identification: 1, TP; 2, caffeine. The chromatographic conditions are as follows: mobile phase of 0.1% acetic acid in water/methanol (70/30, *V*/*V*), flow rate of 1 mL/min, detection wavelength of 280 nm, injection volume is 20 µL, and column temperature is 30 °C.

The extraction selectivity was investigated using TP as analyte and caffeine as interference. The molecular structure of TP is like caffeine, except that TP contains a –NH group at position 9, and caffeine contains –NCH_3_. The sample solution containing the mixture of TP and caffeine with different molar ratios of 1:1, 1:10, and 1:100 was investigated using Fe_3_O_4_-COOH@ZIF-67 nanocomposites as absorbent. It can be seen from Figure 5 that the Fe_3_O_4_-COOH@ZIF-67 can selectively adsorb TP instead of caffeine even if the ratio of interference to target analytes is increased from 1 to 100, manifesting that the Fe_3_O_4_-COOH@ZIF-67 has an excellent selectivity towards TP. Additionally, microporous and mesoporous structures of ZIF-67 form a “trap” to enable the target analytes to easily be captured.

### 2.4. Evaluation of the Adsorption Performance of Fe_3_O_4_-COOH@ZIF-67

#### 2.4.1. Adsorption Isotherms

The static adsorption isotherms of the adsorbent towards TP are exhibited in Figure S2A. The results show that the equilibrium adsorption capacity increases sharply in the range of 100–200 μg/mL, but decreases slowly in 200–300 μg/mL of initial concentration. However, there is an interesting phenomenon that the equilibrium adsorption capacity increases very slowly in the range of 25–100 μg/mL of initial concentration. The parameters fitted by Langmuir and Freundlich adsorption isotherm models are summarized in Table S3. The adsorption of TP on Fe_3_O_4_-COOH@ZIF-67 correlates better with the Langmuir model (R^2^ = 0.993) than the Freundlich model (R^2^ = 0.944), indicating that the adsorption is monolayer chemisorption, the adsorbent surface is homogeneous, and there is no interaction between adsorbed molecules. When reaching equilibrium, the calculated maximum adsorption capacity is as high as 1764 mg/g. Such a high adsorption capacity suggests that Fe_3_O_4_-COOH@ZIF-67 is one of the most effective adsorbents for TP so far. The high adsorption capacity can be ascribed to the high specific surface area, abundant binding sites, and strong multiple adsorption forces of the sorbent to TP, especially the coordination of the Co^2+^ and –NH group on the imidazole ring of TP.

#### 2.4.2. Adsorption Kinetics

The adsorption kinetics of TP on Fe_3_O_4_-COOH@ZIF-67 is shown in Figure S2B. It can be observed from Table S4 that the R^2^ value of the pseudo-first-order (0.8786) model is relatively lower than 0.9774 for the pseudo-second-order model. The calculated adsorption amount is close to the actual values, indicating that the pseudo-second order model can fit better to the experimental data, which is suitable for describing the adsorption process of TP on Fe_3_O_4_-COOH@ZIF-67, showing a rate-limiting step in the adsorption process [16].

### 2.5. Possible Adsorption Mechanism of Fe_3_O_4_-COOH@ZIF-67

**Figure 6:**
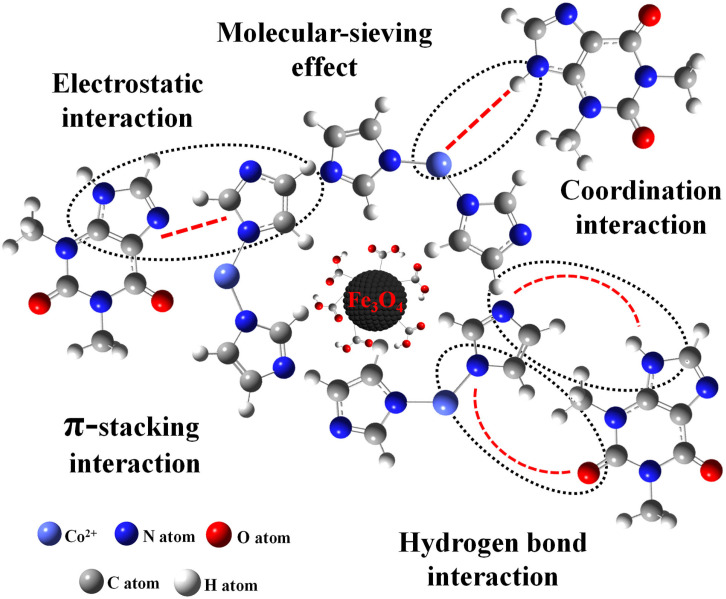
Possible interactions between Fe_3_O_4_-COOH@ZIF-67 and TP, including coordination interaction, hydrogen bond interaction, π-stacking interaction, molecular-sieving effect, and electrostatic interaction.

Figure S3 shows the FT-IR spectra of Fe_3_O_4_-COOH@ZIF-67 before and after the adsorption of TP. The new absorption peak at 2855 cm^−1^ represents the –CH_2_ symmetrical stretching vibration attributed to the methylene from TP. The peak at 2956 cm^−1^ represents the –CH_3_ asymmetrical stretching vibration and 1641 cm^−1^ represents the enhanced –C=O stretching vibration. These results confirm the adsorption of TP. However, the XRD pattern of Fe_3_O_4_-COOH@ZIF-67 (Figure S4) indicates that the crystalline form of the adsorbent was not changed after the adsorption of TP. To understand the possible adsorption mechanism between target analytes and adsorbent as much as possible, seven molecular structurally related compounds were extracted by Fe_3_O_4_-COOH@ZIF-67 nanocomposites, respectively. The adsorption process was performed according to Section 3.5. As shown in Table S5, TP, and theobromine obtain an excellent recovery of 96.71% and 91.17%, respectively. The hypoxanthine has the best recovery (98.89%), but caffeine is close to zero (1.56%). By comparing their molecular structures, all four purine derivatives contain imidazole (Group A), whereas caffeine has a substituent group on N-9 of imidazole moiety. The difference in Group A indicates that coordination interaction between Co^2+^ and –NH from imidazole may play a vital role in the adsorption of analytes, like the formation of ZIF-67. Furthermore, there is no substituent group on N-3 of pyrimidine moiety of hypoxanthine, indicating that –NH on pyrimidine may also coordinate with Co^2+^. This finding can provide guidance for the extraction of pyrimidines by Fe_3_O_4_-COOH@ZIF-67. For further comparison, the compounds containing –COOH (Group B) without purine were also investigated, but they do not have satisfactory results except for vanillic acid (67.36%), and the recovery of ursolic acid and oleanolic acid are 5.88% and 6.25%, respectively. It is noted that hydrogen bond interaction and molecular-sieving effect may also play a role during the adsorption process. Both ursolic acid and oleanolic acid have a larger molecular diameter (12.9 Å and 13.9 Å) than the other six compounds and the sodalite cage diameter of 11.4 Å in ZIF-67 [31], which hinders the target analytes transfer into the micropore of Fe_3_O_4_-COOH@ZIF-67. Furthermore, ursolic acid and oleanolic acid contain plenty of hydrophobic groups such as methyl group, which will further impede their adsorption by the adsorbent. In addition, the imidazole ring can be considered an aromatic compound that can interact with other aromatic compounds via the π-stacking interaction [34]. In short, the mechanisms of interactions (Figure 6) such as coordination interaction, hydrogen bond interaction, π-stacking, and molecular-sieving effect may play an important role in the adsorption of TP by Fe_3_O_4_-COOH@ZIF-67 and may give rise to an ultra-high adsorption capacity.

### 2.6. Reusability and Storage Stability of Fe_3_O_4_-COOH@ZIF-67

**Figure 7:**
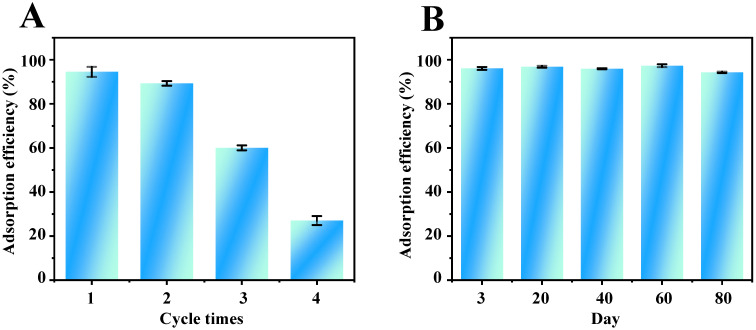
Evaluation of the (**A**) reusability and (**B**) storage stability of Fe_3_O_4_-COOH@ZIF-67.

The reusability of Fe_3_O_4_-COOH@ZIF-67 nanocomposite adsorbent was investigated through four consecutive adsorption–desorption cycles. The results (Figure 7A) show that the adsorption efficiency remained almost unchanged in the first two consecutive cycles, but descended to about 30% in the following two consecutive cycles. Furthermore, the adsorption efficiency of TP varied within 2.5% as shown in Figure 7B after Fe_3_O_4_-COOH@ZIF-67 nanocomposite was stored at room temperature in a dry atmosphere for at least 80 days, indicating that the Fe_3_O_4_-COOH@ZIF-67 adsorbent has good storage stability. The good storage stability of this material is possibly attributed to the ZIF shell and covalent bonding formed by Co–O coordination to achieve stable functionalization.

### 2.7. Method Validation and Real Samples Analysis

The optimum extraction conditions were performed by referring to Section 2.2. The calibration curve of TP in the concentration range of 2.0–100.0 μg/mL was established by HPLC analysis. To evaluate the repeatability of the method, intra-day and inter-day relative standard deviation (RSD) were investigated. The limit of detection (LOD) and limit of quantitation (LOQ) were calculated based on the signal-to-noise ratio (S/N) of 3 and 10, respectively. As shown in Table S6, the determination coefficient (R^2^) is 0.999. The LOD and LOQ are 10.71 and 35.71 ng/mL, respectively. The precision of the method was evaluated through three repeated extractions; the intra-day and inter-day RSD of TP are less than 3.0%.

**Figure 8:**
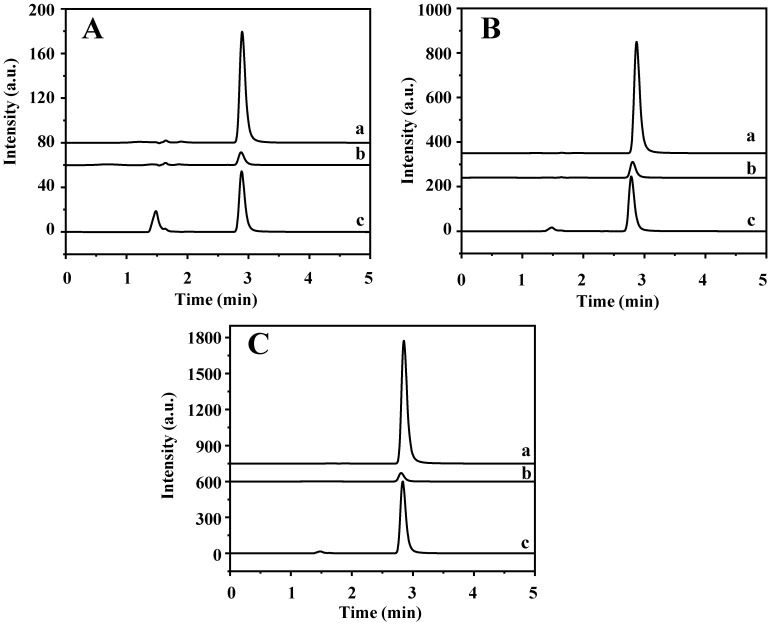
HPLC chromatograms of (a) rabbit plasma sample, (b) supernatant solution after MSPE, and (c) final elution solution being spiked with (**A**) 5 μg/mL, (**B**) 25 μg/mL, and (**C**) 50 μg/mL of TP.

The established method was applied in the determination of TP in four tea samples (jasmine tea, black tea, green tea, and oolong tea) and rabbit plasma to explore its practical applicability. The data are shown in Table 1 and Figure S5. The TP content of jasmine tea, black tea, and green tea are 5.80, 4.31, and 1.53 μg/g, respectively, but oolong tea and rabbit plasma are not detected, which is consistent with the previous reports [2,3]. Furthermore, high levels of theobromine and caffeine that are hundreds and thousands of times higher than TP are found in the four tea samples. In addition, jasmine tea and rabbit plasma samples were spiked with TP at three levels of 5, 25, and 50 μg/mL, respectively. As shown in Table S7 and Figure 8, the recovery of spiked samples is 74.41–86.07%. The results show that this method can be used for the determination of TP in complex real samples.

### 2.8. Comparison with Previously Reported Methods

The comparison of the developed method with the previously reported MSPE, MIP-based, and other extraction methods for the determination of TP are shown in Table 2. The results show that the developed MSPE-HPLC method based on Fe_3_O_4_-COOH@ZIF-67 in this study has many advantages such as rapid adsorption and desorption (within 30 min), ultra-high adsorption capacity (1764 mg/g), and reliable recovery in different matrix samples. Therefore, this established method provides a good choice for the analysis of TP in complex real samples.

## 3. Materials and Methods

### 3.1. Chemicals and Materials

Ferric chloride hexahydrate (FeCl_3_·6H_2_O) was purchased from Mreda Technology Co., Ltd. (Beijing, China). Ethylene glycol (EG), (3-aminopropyl) triethoxysilane (APTES), and sodium phosphate dibasic dodecahydrate (Na_2_HPO_4_·12H_2_O) were purchased from Shanghai Macklin Biochemical Co., Ltd. (Shanghai, China). Sodium acetate anhydrous (NaAC) and ethanol were purchased from Chongqing Chuandong Chemical (Group) Co., Ltd. (Chongqing, China). Polyethylene glycol (PEG-2000) was purchased from Chengdu Kelong Chemical Reagent Factory (Chengdu, China). Methanol, N, N-dimethylformamide (DMF), hydrochloric acid, trisodium phosphate dodecahydrate (Na_3_PO_4_·12H_2_O), ethanolamine, diethanolamine, and triethanolamine were purchased from Chengdu Cologne Chemical Co., Ltd. (Chengdu, China). Tetraethyl orthosilicate, cobaltous nitrate hexahydrate (Co (NO)_3_·6H_2_O), 2-methylimidazole (2-mIm), ammonium hydroxide (AR 25–28%), glutaric anhydride, and theobromine were purchased from Shanghai Aladdin Biochemical Technology Co., Ltd. (Shanghai, China). Polyvinylpyrrolidone (PVP) was purchased from Shanghai Yuanye Bio-Technology Co., Ltd. (Shanghai, China). Theophylline (TP) was purchased from Nantong Feiyu Biotechnology Co., Ltd. (Nantong, China). Caffeine was purchased from Chengdu Prefa Technology Development Co., Ltd. (Chengdu, China). Sodium dihydrogen phosphate (NaH_2_PO_4_) and sodium hydroxide (NaOH) were purchased from Shanghai Titan Scientific Co., Ltd. (Shanghai, China). Cytidine, uridine, inosine guanosine, thymidine, and adenosine were purchased from Sigma Aldrich (Shanghai) Trading Co., Ltd. (Shanghai, China). Ursolic acid (≥98.5%) and oleanolic acid (≥99.0%) were purchased from Chengdu PUSH Bio-technology Co., Ltd. (Chengdu, China). All chemicals were used as received without further purification. Ultra-pure water utilized throughout the experiments was obtained from a water-purification apparatus (ATSelem 1820A, Antesheng Environmental Protection Equipment Co., Ltd., Chongqing, China). All the solvents used in the HPLC analysis such as methanol (MeOH, MW = 32.0) and acetonitrile (ACN, MW = 41.1) were of HPLC-grade and purchased from Adamas Reagent Co., Ltd. (Shanghai, China).

### 3.2. Instruments

The SEM images were obtained using a field-emission scanning electron microscope (FE-SEM) (Quanta 650, FEI, Hillsboro, OR, USA) working at 20 kV. TEM images and element distribution analysis were recorded using a JEM 2100 (JEOL Ltd., Tokyo, Japan) electron microscope working at 200 kV equipped with energy dispersive X-ray spectrometer. FT-IR spectra were taken on a Bruker Tensor 27 spectrometer at 4000–400 cm^−1^ in KBr media. XRD patterns were obtained using X’pert Powder diffractometer (Malvern Panalytical Ltd., Almelo, The Netherlands) with secondary beam graphite monochromated Cu Kα radiation. Nitrogen sorption studies were carried out using a Quadrasorb 2MP (Kantar, Ashland, VA, USA) specific surface and aperture analyzer. Before the adsorption measurements, the samples were activated under vacuum at 120 °C for 24 h. The magnetic properties were measured using a VSM model AGFM/VSM 3886 (Kashan, Iran) at room temperature (about 25 °C) in a magnetic field strength of 2 T. TGA measurements were performed on Switzerland Mettler Toledo TGA2 thermo-analyzer with high-resolution TGA technology MaxRes at a ramp rate of 10 °C/min from 40 °C to 800 °C. Dynamic light scattering particle size analysis and phase analysis light scattering Zeta potential analysis were performed on Brookhaven Instruments Nano-ZS90 Laser nanoparticle analyzer. Blast drying oven (DHG-9015A) and vacuum drying oven (DZF-6012) were purchased from Shanghai Yiheng Scientific Instrument Co., Ltd. (Shanghai, China). Ultrasonic cleaner (KS-3200B) was purchased from Kunshan Jielimei Ultrasonic Instrument Co., Ltd. (Hangzhou, China). Precision balance (ATX124) was purchased from Shimadzu (Tokyo, Japan).

### 3.3. Preparation of Core–Shell-Derived Structural Magnetic Adsorbent

The preparation of core–shell-derived Fe_3_O_4_-COOH@ZIF-67 nanocomposite can be performed according to the following two steps, and the synthesis procedure is shown in Figure 1. Firstly, the glutaric anhydride-functionalized Fe_3_O_4_ nanosphere was prepared through a solvothermal method according to the reference with minor modifications [46]. Typically, FeCl_3_·6H_2_O (2.70 g, 10 mM) was dispersed and dissolved in EG (50 mL) with constant stirring for 30 min. Then, the NaAC (4.50 g) and PEG-2000 (2.60 g) were added into above solution successively with continuous stirring until a transparent solution was obtained. Subsequently, the solution was transferred into a polytetrafluoroethylene (PTFE) autoclave and reacted in the oven at 200 °C for 10 h. After the reaction, the product Fe_3_O_4_ nanospheres were separated by applying an external magnet after being cooled to room temperature (about 25 °C). Finally, the Fe_3_O_4_ was washed with ultra-pure water and ethanol multiple times until the supernatant was colorless, and dried in the vacuum at 60 °C for 6 h. The monodispersed Fe_3_O_4_ nanospheres were modified by APTES and glutaric anhydride [32]. APTES (0.44 mL), glutaric anhydride (0.21 g), and DMF (15 mL) were added into a 100 mL three-necked flask, followed by constant stirring for 3 h at 30 °C. Then, the Fe_3_O_4_ nanospheres (0.30 g), H_2_O (2.25 mL), and DMF (25 mL) were added and vigorously stirred for another 5 h. After being washed with ethanol three times, the glutaric anhydride-functionalized Fe_3_O_4_ nanospheres (Fe_3_O_4_-COOH) were vacuum-dried at 60 °C in an oven overnight.

The Fe_3_O_4_-COOH@ZIF-67 nanocomposite was synthesized through a typical hydrothermal method [42]. In brief, Co (NO)_3_·6H_2_O (0.45 g, 1.55 mM) was dissolved in ultra-pure water (6 mL), solution A was obtained. Then, 2-mIm (5.50 g, 67 mM) was dissolved in ultra-pure water (40 mL), PVP (600 mg), and Fe_3_O_4_-COOH (80 mg) was added successively with ultrasonication for 25 min until a homogeneously dispersed solution B was obtained. Subsequently, solution A was quickly added to solution B within a few seconds. The mixed solution (Co^2+^: 2-mIm: H_2_O = 1:43:1650) was constantly stirred at 42 °C for 30 min, then the resulting dark purple precipitate was collected by applying an external magnet. Finally, the product was washed with ultra-pure water and methanol 3 times and vacuum-dried at 80 °C for 24 h. In addition, pure ZIF-67 was prepared by the same procedure without adding Fe_3_O_4_-COOH nanospheres.

### 3.4. Preparation of Sample Solutions

The stock solution of TP was prepared at a concentration of 2.0 mg/mL by dissolving the reference compound in ultra-pure water and stored at 4 °C, which was prepared and used (diluted to the desired concentrations) within three days. To investigate the applicability of the developed method in the analysis of tea extract and rabbit plasma samples, a spiked recovery study was conducted by triplicate analysis of three spiked concentration levels (5.0, 25.0, and 50.0 µg/mL) of the reference compounds.

Four tea samples (jasmine tea, black tea, green tea, and oolong tea) of different brands were purchased from JD.com, which are from Heshengkang Biotechnology Co., Ltd. (Fujian, China), Shengdayuan Information Technology Co., Ltd. (Shanghai, China), Construction of Tea Refining Plant (Xinyang, China), and Putuo No.1 Branch of Shanghai Jiafeng Tea Co., Ltd. (Shanghai, China), respectively. The tea samples were ground to fine powder. To simulate the preparation of tea by the most commonly used method, an accurately weighed amount of 5.0 g of each individual tea sample was extracted with 100 mL ultra-pure water in a round-bottom flask at 50 °C for 30 min. After being cooled down to room temperature (about 25 °C), centrifugation was performed at 4000 rpm for 6 min. The supernatant was filtered through a 0.45 μm membrane filter (Shanghai Titan Scientific, Shanghai, China) and stored at 4 °C for further MSPE and analysis.

The rabbit plasma (with sodium citrate as the anticoagulant), which is a biological product, was purchased from Shanghai YuanYe Biological Technology Co., Ltd. (Shanghai, China). A total of 100 μL of rabbit plasma was diluted 500 times to 50 mL with ultra-pure water and adjusted to pH 6.0 with 0.1 M HCl and stored at 4 °C for further extraction and analysis.

### 3.5. Procedure of Magnetic Solid-Phase Extraction

In brief, 2.0 mL of the sample solution, containing TP at a concentration of 50 μg/mL, was prepared in a 2 mL centrifuge tube. The pH value of the sample solution was adjusted to 6.0 with HCl (0.5 M). Then, 1.0 mg of prepared Fe_3_O_4_-COOH@ZIF-67 nanocomposites was dispersed in the sample solution with ultrasonication for 5 min and the mixture was shaken on a temperature-controlled air bath thermostatic oscillator (SHZ-82, Jintan Zhengrong Experimental Instrument Factory, Jiangsu, China) at 150 rpm and 30 °C for 20 min to acquire adsorption equilibrium. Subsequently, the nanocomposites were separated by applying an external magnet and the supernatant was discarded. Following, 2.0 mL of Na_3_PO_4_ buffer solution (10 mM, pH 10) was added and stirred at 150 rpm and 30 °C for 5 min. Finally, the resulting desorption solution was separated by the external magnet and filtered through a 0.22 µm membrane filter (Shanghai Titan Scientific Co., Ltd., Shanghai, China) before HPLC analysis. Finally, 2 mL ultra-pure water was added and shaken for about 20 s to wash the adsorbents before next recycling use. The detail chromatographic conditions of HPLC analysis are described in Supplementary Material.

### 3.6. Evaluating the Selectivity of Fe_3_O_4_-COOH@ZIF-67

The selectivity of the prepared Fe_3_O_4_-COOH@ZIF-67 nanocomposites was investigated using TP and caffeine as test compounds with different molar ratios of 1:1, 1:10, and 1:100. Typically, 2.0 mg of Fe_3_O_4_-COOH@ZIF-67 nanocomposite was added to the 2.0 mL mixture solution of TP (2.0 μg/mL) and caffeine (2.0, 20.0, and 200.0 μg/mL, respectively), and extracted and analyzed according to the above procedure.

### 3.7. Evaluation of Adsorption Isotherms and Kinetics

To investigate the adsorption performance of Fe_3_O_4_-COOH@ZIF-67 nanocomposites on TP, static adsorption experiments were carried out at 25 °C by adding 1.0 mg of adsorbents into 8.0 mL solution containing TP with the concentrations of 2, 5, 10, 20, 25, 50, 100, 200, and 300 μg/mL, respectively. The adsorption and desorption processes were performed by referring to Section 3.5. The equilibrium absorption capacity of TP [*Q_e_* (mg/g)] was calculated based on the following Equation (1).

(1) $$Q_{e}=\frac{\left(C_{0}-C_{e}\right)\times V}{m}$$

where *C*_0_ and *C_e_* (μg/mL) represent the initial and equilibrium concentrations of TP, *V* (mL), and *m* (mg) represent the solution volume and the mass of the adsorbent, respectively.

The adsorption isotherm was used to describe the relationship between the equilibrium adsorption capacity and the equilibrium concentration under certain adsorption temperature [47]. To determine the adsorption capacity and adsorption mechanism for TP of Fe_3_O_4_-COOH@ZIF-67, the Langmuir [Equation (2)] and Freundlich [Equation (3)] were used to fit the experimental data as follows, respectively.

(2) $$\frac{1}{Q_{e}}=\frac{1}{Q_{max}{\times K}_{L}}\times \frac{1}{C_{e}}+\frac{1}{Q_{max}}$$

(3) $$lnQ_{e}=\frac{1}{n}\times lnC_{e}+lnK_{F}$$

where *Q_max_* is the maximum adsorption capacity (mg/g), *K_L_* (L·mg^−1^), and *K_F_* [(L·mg^−1^)^1/*n*^] are Langmuir and Freundlich adsorption constants, respectively. The 1/*n* represents the intensity of adsorption.

To gain insight into the adsorption kinetics of TP by Fe_3_O_4_-COOH@ZIF-67, the dynamic adsorption experiment was carried out at 30 °C by adding 2.0 mg of adsorbents into 2.0 mL solution containing 50 μg/mL of TP. The adsorption and desorption processes were performed according to Section 3.5., and the pseudo-first-order [Equation (4)] and pseudo-second-order [Equation (5)] kinetic models were used to fit the experimental data as follows, respectively.

(4) $$ln\left(1-\frac{Q_{t}}{Q_{e}}\right)=-k_{1}\times t$$

(5) $$\frac{t}{Q_{t}}=\frac{1}{k_{2}\times Q_{e}^{2}}+\frac{1}{Q_{e}}\times t$$

where *Q_t_* (mg/g) is the adsorption capacity at time *t* (min), *k*_1_ (min^−1^) and *k*_2_ (g·mg^−1^·min^−1^) are adsorption rate constants.

## 4. Conclusions

A single-step MOF-coated core–shell-derived Fe_3_O_4_-COOH@ZIF-67 nanocomposite was prepared via a solvothermal method and employed as the MSPE adsorbent for TP. The coordination interaction between undercoordinated Co^2+^ on ZIF-67 and –NH from imidazole and other interactions result in an ultra-high adsorption capacity for TP. Fe_3_O_4_-COOH@ZIF-67 nanocomposites can be quickly dispersed well in an aqueous solution and separated by an external magnet. The entire MSPE process can be finished within 30 min. Combined with HPLC analysis, the developed pretreatment method is highly efficient and sensitive to TP. Despite its obvious advantages, the method does not have a relatively high recovery. Thus, further studies can be conducted focusing on the enhancement of the recovery of analytes such as TP.

## Figures and Tables

**Table 1:** Determination of TP in real samples by MSPE-HPLC (mean ± SD, *n* = 3).

Sample	TP (μg/g)	RSD (%) ^a^
jasmine tea	5.80 ± 0.20	3.4
black tea	4.31 ± 0.11	2.5
green tea	1.53 ± 0.02	1.3
oolong tea	ND _b_	ND
rabbit plasma	ND	ND

^a^ Relative standard deviation. ^b^ Not detected.

**Table 2:** Comparison of the developed method with previous reported methods for the determination of TP.

Extraction Method	Analytes	Extraction Time (min)	Capacity (mg/g)	Recovery (%)	Application	Ref.
MIP/SPE	TP	>250	0.167	79–83	human serum	[14]
MIP/MSPE	TP, TB	>270	5.07, 4.87	87.51, 92.27	green tea	[15]
MIP/MSPE	TP, CA	>720	3.301, 2.436	98.7–100.8, 98.3–100.2	green tea	[45]
MSPE	TP	>100	-	91.2–100.4	rabbit and rat plasma	[17]
MSPE	TP	120	146	54.80–77.90	plasma and milk	[16]
PT-SPE	TP	>400	93.25	82.83–93.08	tea	[11]
UA-SEME	TP, caffeine	10	-	96.3–104.0, 98.8–102.0	human plasma and cocoa powder	[12]
MSPE	TP	30	1764	74.41–86.07	four types of tea and rabbit plasma	this work

-, Not mentioned; MIP, molecularly imprinted polymer; TB, theobromine; CA, Chlorogenic acid; PT-SPE, Pipette-tip solid-phase extraction; UA-SEME, ultrasound-assisted surfactant-enhanced emulsification microextraction.

## Data Availability

The data presented in this study are contained within the article.

## Supplementary Materials

The following supporting information can be downloaded at: https://www.mdpi.com/article/10.3390/molecules28145573/s1. Table S1. Structural parameters of Fe_3_O_4_-COOH@ZIF-67; Table S2. The basic properties of TP; Table S3. Adsorption isotherm parameters of TP on Fe_3_O_4_-COOH@ZIF-67 nanocomposites; Table S4. The kinetics of TP adsorption on Fe_3_O_4_-COOH@ZIF-67 nanocomposites; Table S5. The recoveries of compounds adsorbed by the Fe_3_O_4_-COOH@ZIF-67 nanocomposites (*n* = 3); Table S6. Linear regression data and precision for the determination of TP (*n* = 3); Table S7. Spiked recoveries of TP in real samples analyzed by MSPE-HPLC (mean, *n* = 3); Table S8. The adsorption efficiencies of six nucleosides adsorbed by the Fe_3_O_4_-COOH@ZIF-67 nanocomposites (*n* = 3); Figure S1. Effects of pH of desorption solution (A), volume of desorption solution (B), and concentration of PO_4_^3−^ (C) on the desorption efficiency of TP; Zeta potential analysis of Fe_3_O_4_-COOH@ZIF-67 in different pH value (D); Figure S2 Adsorption isotherm (A), adsorption kinetics (B), Langmuir (C), and Freundlich plots of the isotherm (D) for TP adsorption on Fe_3_O_4_-COOH@ZIF-67; Figure S3. The FT-IR spectra of Fe_3_O_4_-COOH@ZIF-67 before and after adsorbed TP; Figure S4. The XRD patterns of Fe_3_O_4_-COOH@ZIF-67 before and after adsorbed TP; Figure S5. The representative HPLC chromatograms of three mixed standard solutions of methylxanthines (a), final elution solution of jasmine tea (b), black rea (d), green tea (c), and oolong tea (e) after MSPE. 1, theobromine; 2, TP; 3, caffeine.

## Sample Availability

Samples of the compounds are available from the authors.
