# Supplementary material for: Ultra-High Adsorption Capacity of Core–Shell-Derived Magnetic Zeolite Imidazolate Framework-67 as Adsorbent for Selective Extraction of Theophylline
Source: Molecules. 2023 Jul 21;28(14):5573. doi: 10.3390/molecules28145573 (PMC10383973; doi:10.3390/molecules28145573)
Supplement: Supplementary file 1 [file molecules-28-05573-s001.zip › molecules-2482440-supplementary.pdf]

Electronic Supplementary Material

Ultra-High Adsorption Capacity of Core–Shell -Derived Magnetic Zeolite Imidazolate Framework-67 as Adsorbent for Selective Extraction of Theophylline

Ling-Xiao Chen [1], Shi-Jun Yin [1], Tong-Qing Chai [1], Jia-Li Wang [1], Guo-Ying Chen [1], Xi Zhou [1], Feng-Qing Yang [1*]

[1] School of Chemistry and Chemical Engineering, Chongqing University, Chongqing 401331, PR China

*Prof. Dr. Feng-Qing Yang, School of Chemistry and Chemical Engineering, Chongqing University, Chongqing 401331, China.

Phone number: +8613617650637. E-mail: fengqingyang@cqu.edu.cn

Supplementary Methods

Chromatographic conditions of HPLC analysis

HPLC analysis was performed on an Agilent 1260 Series liquid chromatography system (Agilent Technologies, Palo Alto, California, USA), which is equipped with a vacuum degasser, a binary pump, an auto-sampler, and a diode array detector and is controlled by the Agilent ChemStation software. An Agilent ZORBAX SB-C18 column (150 × 4.6 mm i.d., 5 μm) and a pre-column (ZORBAX SB-C18 guard column, 12.5 × 4.6 mm i.d., 5 μm) were employed to separate target theophylline (TP) and caffeine. The mobile phase consisted of 0.1% acetic acid in water (A) and methanol (B) was at the flow rate of 1.0 mL/min, detection wavelength was 280 nm, injection volume was 10 μL and column temperature was at 30 °C. For the analysis of reference compounds, the isocratic elution of A: B = 30:70 (*v*/*v*) was used. For the analysis of tea extract and rabbit plasma, the gradient elution was performed as follows: 0–6 min, 20–36% B; 6–12 min, 36–60% B; 12–13 min, 60% B; 13–14 min, 60–20% B; and finally, 20% B for 3 min.

Supplementary Tables

Table S1. Structural parameters of Fe_3O_4-COOH@ZIF-67

	S_{BET} (m^2/g)	V_{pore} (cm^3/g)	D_{pore} (nm)
Fe_3O_4-COOH@ZIF-67	1465.3	0.5855	1.5984

Table S2. The basic properties of TP

Compounds	Molecular Weight	pKa[a]	Log P[b]	H Acceptors	H Donors
TP	180.16	1.64±0.70<br>8.60±0.50	0.276±0.296	6	1

[a] Data is obtained from SciFinder

[b] Temperature 25 °C

Table S3. Adsorption isotherm parameters of TP on Fe_3O_4-COOH@ZIF-67 nanocomposites

**Analyte**	**Langmuir**			**Freundlich**		
	Q_{max} **(mg/g)**	K_L **(mL/mg)**	$\mathbf{R^2}$	K_F **($mg^{(1-1/n)}L^{(1/n)}g^{-1}$)**	n	$\mathbf{R^2}$
TP	1764	3.518×10^{-3}	0.993	8.299	1.0201	0.944

Table S4. The kinetics of TP adsorption on Fe_3O_4-COOH@ZIF-67 nanocomposites

T (K)	Pseudo-first-order dynamics model			Pseudo-second-order dynamics model		
298	Q_e (mg/g)	K_1 (min^{-1})	R^2	Q_e (mg/g)	K_2 ($g \cdot mg^{-1} \cdot min^{-1}$)	R^2
	48.42	0.3172	0.8786	50.50	0.0182	0.9774

Table S5. The recoveries of compounds adsorbed by the Fe_3O_4-COOH@ZIF-67 nanocomposites (*n*=3)

No.	Group	Compound	Structural formula	Molecular diameter [a]	Recovery (%) [b]
1	A	TP	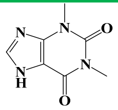	7.5 Å	96.71
2	A	theobromine	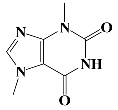	7.6 Å	91.17
3	A	caffeine	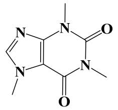	7.6 Å	1.56
4	A	hypoxanthine	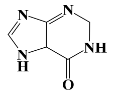	6.4 Å	98.89
5	B	vanillic acid	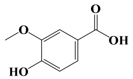	8.0 Å	67.36
6	B	ursolic acid	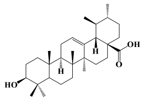	12.9 Å	5.88
7	B	oleanolic acid	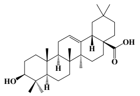	13.9 Å	6.25

[a] Data was simulated by ChemDraw19.0

[b] Data was obtained at optimum

Table S6. Linear regression data and precision for the determination of TP (*n*=3)

Analytes	Liner regression					Precision	
	Calibration curve	R^2	Liner range (μg/mL)	LOD (ng/mL)	LOQ (ng/mL)	Intra-day RSD (%)	Inter-day RSD (%)
TP	y=137.27x-8.2015	0.9999	2.00–100	10.71	35.71	0.58	2.72

Table S7. Spiked recoveries of TP in real samples analyzed by MSPE-HPLC (mean, *n*=3)

Sample	Spike level (μg/mL)	Recovery (%)	RSD (%)
jasmine tea	5	79.94	1.80
	25	76.92	3.83
	50	79.92	0.82
rabbit plasma	5	81.68	1.46
	25	74.41	1.30
	50	86.07	0.81

Table S8. The adsorption efficiencies of six nucleosides adsorbed by the Fe_3O_4-COOH@ZIF-67 nanocomposites (n=3)

Nucleosides	Structural formula	Adsorption efficiency (%)	RSD (%)
cytidine	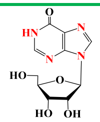	1.93	0.83
uridine	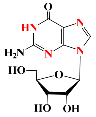	48.68	2.54
inosine	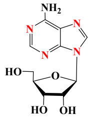	36.95	3.21
guanosine	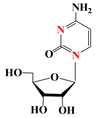	36.95	1.08
thymidine	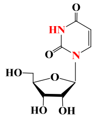	41.83	1.58
adenosine	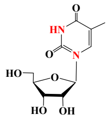	3.55	0.56

Supplementary Figures

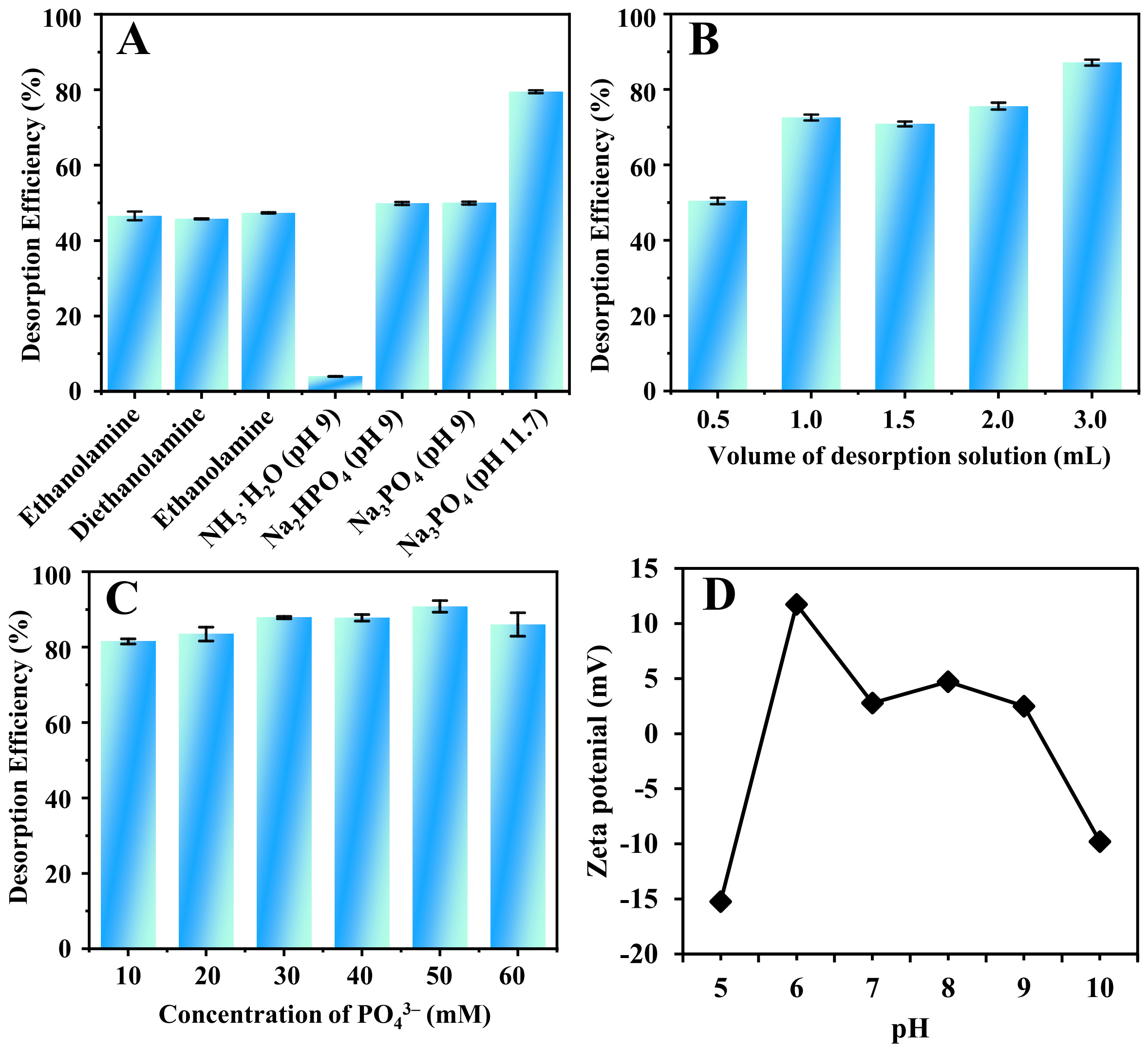

Figure S1. Effects of pH of desorption solution (A), volume of desorption solution (B), and concentration of PO_4^{3-} (C) on the desorption efficiency of TP; Zeta potential analysis of Fe_3O_4-COOH@ZIF-67 in different pH value (D).

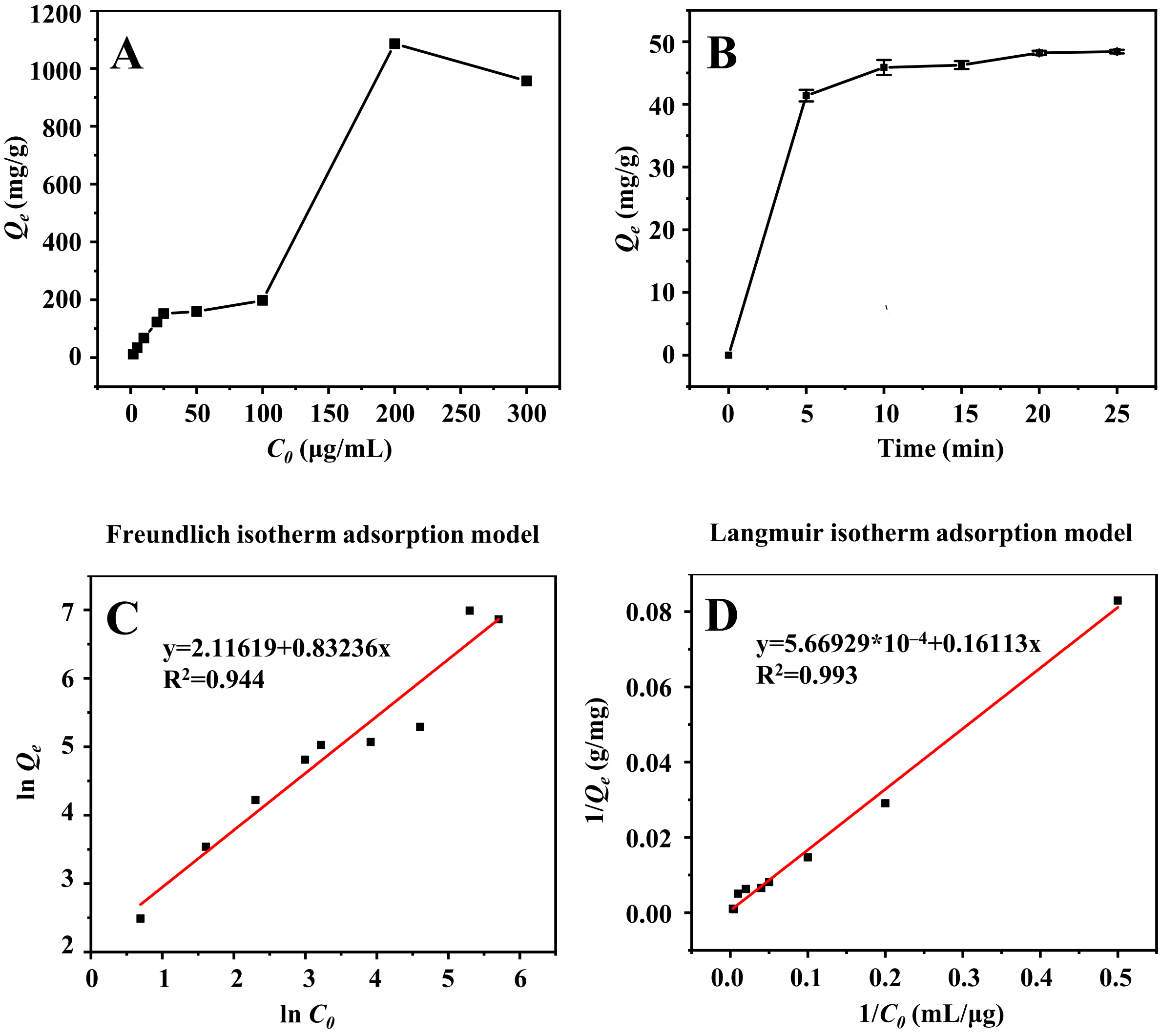

Figure S2. Adsorption isotherm (A), adsorption kinetics (B), Langmuir (C), and Freundlich plots of the isotherm (D) for TP adsorption on Fe_3O_4-COOH@ZIF-67.

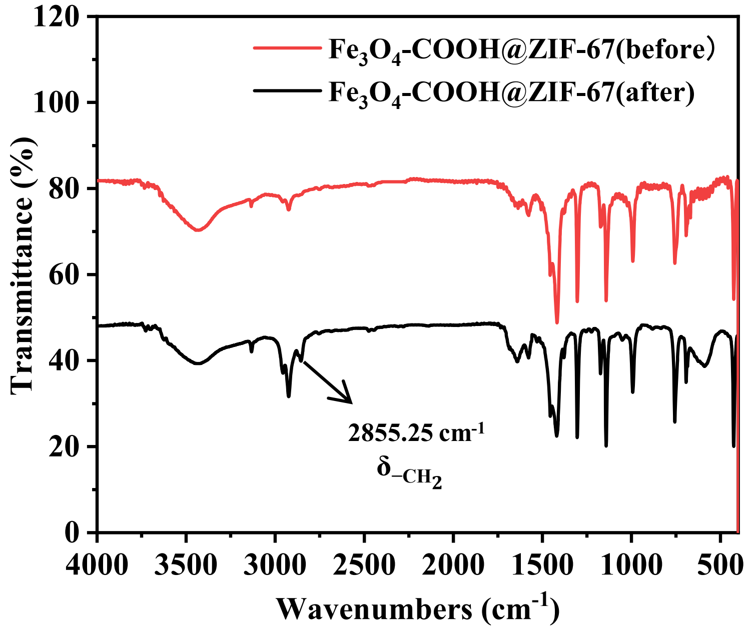

Figure S3. The FT-IR spectra of Fe_3O_4-COOH@ZIF-67 before and after adsorbed TP.

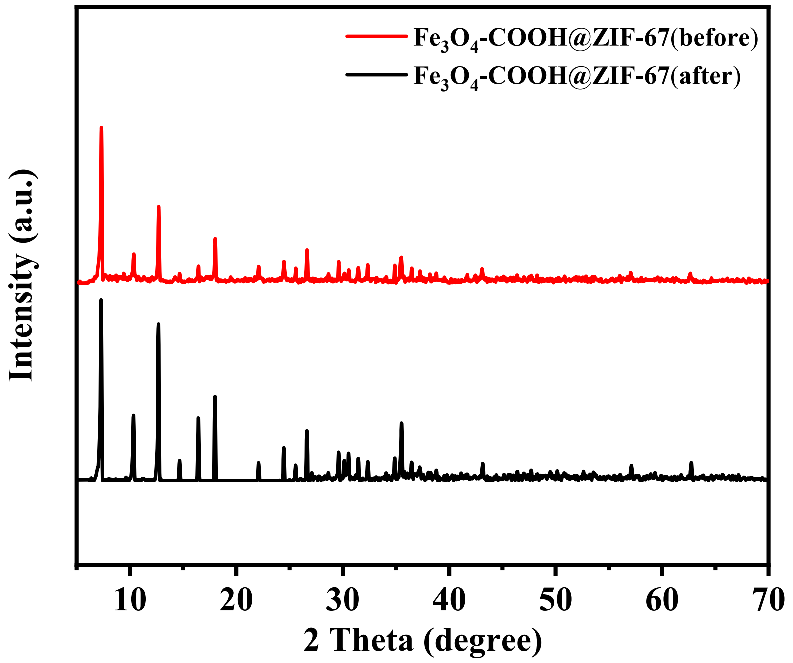

Figure S4. The XRD patterns of Fe_3O_4-COOH@ZIF-67 before and after adsorbed TP.

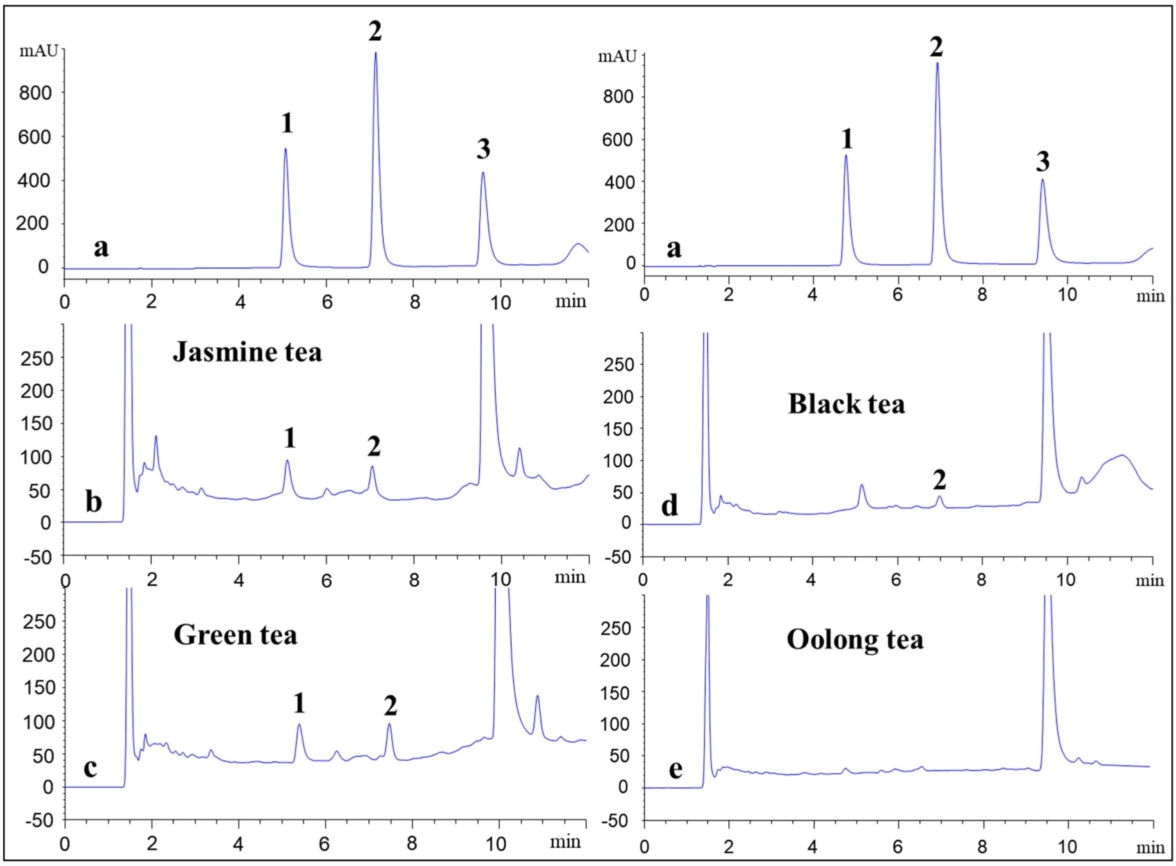

Figure S5. The representative HPLC chromatograms of three mixed standard solutions of methylxanthines (a), final elution solution of jasmine tea (b), black rea (d), green tea (c), and oolong tea (e) after MSPE. 1, theobromine; 2, TP; 3, caffeine.
